# The role of natural compounds in modulation of small extracellular vesicle biogenesis and lipid metabolism in cancer

**DOI:** 10.3389/fmolb.2025.1727274

**Published:** 2025-11-13

**Authors:** Gwan Yong Lim, Laksheetha Santhakumar, Magdalena Mielczarek-Puta, Michał Skrzycki

**Affiliations:** 1 Chair and Department of Biochemistry, Faculty of Medicine, Medical University of Warsaw, Warsaw, Poland; 2 Student Scientific Club Explore, Chair and Department of Biochemistry, Faculty of Medicine, Medical University of Warsaw, Warsaw, Poland

**Keywords:** small extracellular vesicle, natural compound, lipid metabolism, tumor progression, cancer treatment

## Abstract

Small extracellular vesicles (sEVs) are vesicles of 15–150 nm in diameter secreted by cells and contain biological compounds that aid in cell growth, proliferation, and communication. Over the years, their role in oncogenesis has become prominent, especially in modulating the tumor microenvironment, facilitating epithelial-mesenchymal transition, and promoting metastasis. These oncosomes serve as unique diagnostic markers that can be used to detect specific types of cancer. Their stable lipid bilayer is composed of various classes of lipids, including phosphatidylserine, sphingomyelin, ceramides, and sterols. Alterations in the lipid profile of sEVs have been found in various chronic diseases, including cancers, making them suitable biomarkers and therapeutic targets. Natural compounds (NCs) derived from plants and microbes exhibit antitumorigenic properties. They have been recognized in contemporary medicine for their capacity to modulate sEV synthesis, secretion, and composition. However, there is limited research on the effects of NCs on the lipid panel of extracellular vesicles, as most studies have focused on proteins and microRNAs. Considering that NCs can influence key regulatory enzymes involved in lipogenesis and degradation, this suggests a potential impact on the lipid composition of sEVs. Therefore, we summarized the direct effects of NCs on sEVs and lipid-related enzymes, highlighting the potential for natural compound-mediated lipid modulation in sEVs.

## 1 Introduction

Extracellular vesicles (EVs) are membrane-bound, spherical particles containing functional cargo: proteins, nucleic acids, and lipids. They play crucial roles in intercellular and interorgan communication. EVs are classified into different subtypes, based on their size and biological function. The formation and release of EVs are controlled by various mechanisms, primarily the endosomal sorting complex required for transport (ESCRT). ESCRT is a highly conserved molecular machinery composed of five different ESCRT complexes (ESCRT-0, -I, -II, -III and Vps4) (Gurung et al., 2021). ESCRT-0 recognizes the ubiquitinated cargoes and recruits them to endosomal microdomains, which are mediated by binding to 3-phosphoinosides. ESCRT-I and ESCRT-II subsequently drive inward budding of the endosomal membrane, forming intraluminal vesicles inside multivesicular bodies. Next, ESCRT-III transiently assembles on endosomal membrane for the final step of vesicle formation. ESCRT-III is believed to have two major functions: cargo sequestration and membrane budding and scission (Schmidt and Teis, 2012; Colombo et al., 2013). Along with the ESCRT-dependent pathway, lipids also act an essential role in EVs' formation, secretion, and stability maintenance. Ceramide, an essential lipid involved in cellular signaling, has been shown to trigger budding of exosomes without the ESCRT system (Trajkovic et al., 2008). Other lipids (cholesterol, sphingomyelin, and phosphatidylserine) have also been shown to participate in the formation, secretion, signaling, and uptake of exosomes. This highlights the role of lipids in EVs' formation and secretion (Record et al., 2014).

According to the International Society for Extracellular Vesicles guideline, small extracellular vesicles (sEVs) refer to vesicles with a diameter smaller than 200 nm (Théry et al., 2018). sEVS are released from cells and are often found to contain biologically active compounds that trigger transformation in recipient cells either through paracrine signaling or released directly into the bloodstream, encouraging intercellular communication. The development of EV-based biomarkers depending on the various types of cargos they carry can be beneficial for diagnosing diseases, such as cancer, neurological diseases and metabolic diseases. Beyond their diagnostic potential, sEVs also actively participate in tumor progression by transporting oncogenic proteins, lipids, and genetic materials, resulting in metabolic modifications in microenvironment.

Natural compounds are bioactive substances naturally found in organisms. They are widely used in traditional medicine, particularly in East Asia, and are now increasingly recognized for their anti-cancer potential.

This mini review summarizes the role of sEVs in development of cancer and its therapeutic potential, while highlighting NCs as modulators that influence vesicle biogenesis, composition, and function of sEVs. Moreover, sEVs themselves serve as promising drug delivery tools, enhancing cellular uptake while decreasing side effects due to low specificity.

## 2 Role of sEVs in development of cancer

Cancerous cells prompt to release sEVs containing pro-tumorigenic biological compounds, resulting in induction of oncogenesis in recipient cells and promote epithelial to mesenchymal transitions (Peinado et al., 2012; Zhang and Grizzle, 2011). This includes RAS, KRAS, EGFR, mutant EGFR variant III, heat shock proteins (HSP), chaperonins, matrix metalloproteinases (MMP), endoplasmins, integrins, epithelial CAMs and PD-L1. Such oncogenic sEVs are often known as oncosomes, which result in the formation of tumor microenvironment and maintenance of premetastatic niche (Kalluri and LeBleu, 2020).

Macrophages localized in the tumor microenvironment termed tumor associated macrophages (TAMS) are key players in cancer development. M2 phenotype TAMS secretes exosomes that contribute to metastasis of GC cells via the ApoE-activating PI3-Akt signaling pathway leading to favorable cytoskeleton remodeling to support migration (Qian and Pollard, 2010).

The phospholipid phosphatidylserine (PS) is present abundantly in the inner leaflet of the cell membrane and is also found primarily in the sEVs released as observed in *ex vivo* tumoroid cells which mimic mammalian tumors and their environment (Skotland et al., 2020). sEVs mediates transfer of multifunctional proteins like MMP and HSP. MMP are involved in cancer stem cell associated premetastatic niche formation, specific endopeptidases like MMP-3 are involved in transcellular signaling which promotes tumorigenesis via activation of the connective tissue growth factor when taken up by recipient cells. Meanwhile HSP90 is mainly involved in intracellular signaling via sEVs and is abundant in highly metastatic cancers (Shimoda and Khokha, 2017). HSP90 alpha specifically is found to promotes EMT. In addition, chaperonins such as HSP60 are found abundantly in gliomas and cancers of the large bowel.

## 3 Natural compounds and extracellular vesicles: modulators of oncogenic vesicles and tools for drug delivery

Natural compounds (NCs) are produced naturally by living organisms and have been broadly used in tranditional medicine. Studies have shown that NCs plays an essential role in regulating hypertension, inflammation, pain, and cancer progression (Seca and Moujir, 2020). Their anticancer mechanisms includes facilitating lipid peroxidation, triggering cell deaths (apoptosis and ferroptosis), and reduction of sEV oncogenic cargo production (Tang et al., 2024; Fyfe et al., 2023). Additionally, NCs have been reported to influence lipid-metabolizing enzymes that could affect vesicular formation (Table 1). Table 1 summarizes the effects of natural compounds on lipid metabolism, extracellular vesicles, and their overall regulatory effects.

**TABLE 1:** Natural compounds modulating extracellular vesicles (EVs) and lipid metabolism, with overall regulated effects on lipid related pathway.

Compound	Primary effects on EVs	Effects on lipid metabolism	Regulatory effect	Reference
Manumycin A	Decreased exosome secretion; ESCRT inhibition; Ras/Raf/ERK1/2 blockade; decreased hnRNP H1	FTase inhibition; decreased Ras/Rheb prenylation; downregulation ofmTOR and MAPK signaling; reduced lipogenesis	Downregulated	Cho et al. (2015), Datta et al. (2017)
Cannabidiol	Reduced exosome and microvesicle release; increased miR-126, decreased miR-21; decreased prohibitin expression	Decreased FAS activity; altered sphingolipid metabolism	Downregulated	(Kosgodage et al., 2018; Kosgodage et al., 2019; Fu et al., 2025; Konstantynowicz-Nowicka et al., 2025)
Resveratrol	Reduced exosome secretion (via decreased Rab27a in Huh7 cells); increased CD63 and Ago2 in COLO320 cells; decreased eIF2α in COLO741 cells	Decreased FAS,SCD1, and ACC expression; reduced *de novo* lipogenesis	Downregulated	(Tong et al., 2024; Becer et al., 2024; Ardid-Ruiz et al., 2019; Gimeno-Mallench et al., 2019)
Honokiol	-	Decreased SREBP-1c maturation,SCD1, andFAS; Increased AMPK phosphorylation	Mixed (inhibits lipogenesis, activates AMPK)	Seo et al. (2015)
Dihydroarteminisin	-	Decreased expression of c-Myc,ACC,FAS,CPT-1, and MCAD	Downregulated	Hu W et al. (2021)
Curcumin	Increased exosomal TCF21; enhanced exosome/microvesicle release	Increased ceramide synthesis; Reduced endolysosomal lipid accumulation	Upregulated	(Taverna et al., 2015; García-Seisdedos et al., 2020)
EGCG (Epigallocatechin Gallate)	Increased exosomal miR-16 expression	Decreased FAS and ACC expression; IncreasedCPT-1 activity; Reduced lipid droplet formation	Mixed (downregulates lipogenesis, upregulates β-oxidation	(Jang et al., 2013; Wang et al., 2021)

ACC, acetyl-CoA carboxylase; CPT-1, Carnitine–palmitoyltransferase-1; ESCRT, endosomal sorting complex required for transport; FAS, fatty acid synthase; FTase, farnesyltransferase; MCAD, Medium-chain acyl-CoA, dehydrogenase; SCD1, stearoyl-CoA, desaturase-1; SREBP-1c, sterol regulatory element-binding protein-1c; TCF, transcription factor.

### 3.1 Natural compound with direct effects on extracellular vesicles

#### 3.1.1 Manumycin A

Manumycin A (MA) is an antibiotic derivative of *streptomyces* species with well-established antitumor effects (Cho et al., 2015). MA significantly reduced of exosome secretion (10 fold) in castration-resistant prostate cancer (CRPC) cells by shutting down endosomal sorting complex required for transport and inhibit Ras/Raf/ERK1/2 signaling and hnRNP H1 expression, while normal prostate cells were not affected (Datta et al., 2017). Suppression of Ras pathway also caused CRPC to become more sensitized to enzalutamide, making MA a potential candidate for CRPC treatment.

#### 3.1.2 Cannabidiol

Cannabidiol (CBD), a phytocannabinoid found in *Cannabis sativa*, has been reported to directly modulate exosome and microvesicle release in multiple cancer cell lines including prostate cancer (PC3), hepatocellular carcinoma (HEPG2) and breast adenocarcinoma (MDA-MB-231) (Kosgodage et al., 2018). In glioblastoma multiforme cells, CBD reduced exosome release and altered the level of microRNA (increase in miR-126 and decrease in miR-21). Since level of miR-126 directly corresponds to the survival rate and duration in glioblastoma multiforme patient, CBD-mediated microRNA may act as promising therapeutic agent. CBD also inhibits prohibitin, which is known to be a chaperoning protein associated with chemoresistance (Kosgodage et al., 2019). Besides direct effect of CBD on exosomes and its vesicular content, camel milk-derived exosomes enhanced cytotoxic efficacy of CBD against doxorubicin-resistance breast cancer by improving bioavailability, making sEVs a good candidate for drug delivery (Aare et al., 2024).

#### 3.1.3 Resveratrol

Resveratrol is a natural polyphenol with antioxidant characteristics. It has been used to prevent multiple chronic diseases, including cancer (Ko et al., 2017). The study by Tong et al. (2024) showed that resveratrol blocked exosome secretion by downregulating Rab27a in Huh7 cells. The declines in exosome secretion further results in antiproliferation and decreased migration ability in Huh7 cells. Resveratrol also increases CD63 and Ago2 levels in COLO320 (primary colorectal adenocarcinoma), while reducing eIF2α in COLO741 cell line (metastatic colorectal adenocarcinoma) (Becer et al., 2024).

##### 3.1.4 Honokiol

Honokiol is a vital bioactive compound isolated from genus *Magnolia* (Banik et al., 2019). In preliminary research, honokiol demonstrated anticancer activity across multiple cancer models. It has been shown to increase bioavailability after sonication with exosomes secreted by mesenchymal stem cells, facilitating the delivery to cancer cells by increasing cellular uptake, while reducing the toxicity to normal cells (Kanchanapally et al., 2020). In addition to its role as an exosome-loaded therapeutic agent, honokiol was identified as P-glycoprotein (P-gp) inhibitor. It was shown by using inside-out orientation extracellular vesicles encapsulating magnetic nanoparticles (IOVMNPs) as a novel screening platform (Zhang et al., 2023). The direct effects of honokiol on sEVs remain to be explored.

##### 3.1.5 Dihydroartemisinin

Dihydroartemisinin (DHA), the active metabolite of artemisinin, is a sesquiterpene lactone well known for its potent antimalarial activity. DHA was reported to downregulate the expression of HSP70 in prostate cancer (PC3) cells (Kong et al., 2019). As HSP70 is a common marker associated with small extracellular vesicles (sEVs), this may suggest that DHA may lower oncosome formation, blocking intracellular communication of tumor promoting factors. Nevertheless, the exact effect of DHA remains unknown. Kumar et al. (2025) showed that exosomal formulation of DHA improved in the efficacy of DHA in melanoma cell model compared to DHA alone.

### 3.2 Natural compound modulating lipid-metabolizing enzymes relevant to EV synthesis

Although few studies have directly explored the NCs effects on EVs’ lipid composition, many of them are known to regulate key enzymes related to lipid-synthesis or degradation, which are essential for vesicle formation. Manumycin A inhibits farnesyltransferase (FTase), a protein-modifying enzyme that attaches hydrophobic lipid tail to the protein. FTase is crucial for signaling and trafficking of vesicles, by farnesylating Ras and Rheb, thereby modulating the mTOR and MAPK. These pathways indirectly effect ESCRT-dependent EV biogenesis, which is needed to vesicular excretion. Cannabidiol decreases fatty acid synthase (FAS) activity and alters sphingolipid metabolism (Fu et al., 2025; Konstantynowicz-Nowicka et al., 2025). Resveratrol downregulates fatty acid synthase (FAS) and stearoyl-CoA desaturase-1 (SCD-1), suppressing *de novo* lipogenesis (Ardid-Ruiz et al., 2019). Acetyl-CoA carboxylase (ACC) and its products are also suppressed after resveratrol treatment (Gimeno-Mallench et al., 2019). Honokiol inhibits sterol regulatory element-binding protein-1c (SREBP-1c) maturation and reduces SCD-1 and FAS expression (Seo et al., 2015). Dihydroartemisinin downregulates c-Myc, an oncogenic driver in various cancers. The reduction in c-Myc expression resulted in decreased formation of ACC, FAS, carnitine–palmitoyltransferase-1 (CPT-1), and medium-chain acyl-CoA dehydrogenase (MCAD) (Hu X et al., 2021). In summary, these enzymes are key regulators of lipid homeostasis, suggesting that NCs indirectly affect EV biogenesis and biological function by altering lipid metabolism.

## 4 Therapeutic applications and challenges

### 4.1 Inhibition of sEV secretion pathways

The release of sEV from the cells is dependent on two pathways. (ESCRT-dependent and ESCRT-independent). ESCRT-independent pathway uses neutral sphingomyelinase 2, which hydrolyzes sphingomyelin to produce active ceramides. Since sEVs are also involved in transmission of pathogenic diseases, drugs targeting the cellular pathways involved in the secretion of sEV are of importance. Manumycin A and tipifarnib inhibit ESCRT and interfere with sEVs release. Treatment with tipifarnib reduced the release of sEVs in renal cell carcinoma patients, while manumycin also reduced the release of sEVs in patients diagnosed with castration-resistant prostate cancer.

### 4.2 Oncogenic miRNA

Cancer cells release sEVs containing oncogenic miRNAs. MiRNAs such as miRNA181a and miRNA −199a regulate the progression of glioma and increases the sensitivity of glioma cells to temozolomide respectively. They are currently being explored as novel therapeutic strategies when delivered via sEVs (Li et al., 2019). Among these, miRNA-199a-3p suppresses glioma cell proliferation by targeting the AKT/mTOR signaling pathway, a key regulator of cell growth, survival, and metabolism in cancer (Shen et al., 2015). Thymoquinone has shown to upregulate microRNA-199a-3p expression in Human A549 lung carcinoma cells, making it a potential adjuvant compound for miRNA based therapy (Khan et al., 2025).

### 4.3 Biomarker and function of cancer-derived sEVs

Women remain at an increased risk of developing breast cancer for up to 5 years after pregnancy. Mammary gland cell–derived sEVs present in breast milk contain TGF-β2, which promotes EMT in both MCF-7 and MCF-10A breast cancer cells. Patients with poor prognosis of BC had sEVs with mRNAs coding for NANOG, NEUROD1, HTR7, KISS1R, and HOXC6 amongst over 100 miRNAs that were identified as BC biomarkers. sEVs are stable due to their lipid bilayer configuration, typical characteristic of an exosome (Loric et al., 2023).

In prostate cancer patients, cancer cells secrete sEVs containing adrenomedullin which upregulates fatty acid oxidation via ERK1/2 and p38 MAPK axis contributing to the early weight loss associated with prostate cancer patients (Sagar et al., 2016). In addition, cancer-associated cachexia can also secrete adipokines and adipocytokines to aid in tumor cell growth and development. This elucidates the importance of sEVs in cancer and how therapeutic intervention targeting sEVs can be very beneficial in treatment of cancer. Lysophosphatidylcholine, phosphatidylcholine, and phosphatidylethanolamine are also biomarkers for pancreatic cancer. Contrasting this, there is decreased levels of sphingosine-1-phosphate, elaidic carnitine and palmitoyl carnitine found in patients suffering from melanoma and hepatocellular carcinoma (Bland et al., 2018; Zhou et al., 2012).

### 4.4 Challenge of targeted delivery using engineered exosomes

One of the major drawbacks in developing therapeutic treatment using exosomes is targeting drug delivery to specific tissues instead of all tissues. Considering non-specific uptake of engineered exosomes being taken up by the liver, this may exert great stress on its xenobiotic system. Current research is aiming to solve this problem by focusing on membrane proteins incorporated in exosomes and attention has been drawn to LAMP2B. LAMP2B isoform is a transmembrane protein abundantly present in exosomes and when utilized as target for fusion with specific tissue types, success has been documented (Hung and Leonard, 2016). IL-3 and LAMP2B fused exosomes loaded with imatinib were able to selectively deliver medication to chronic myeloid leukemia and inhibit tumorigenesis (Bellavia et al., 2017). Similar engineered exosomes were also used in treatment of glioblastoma, anaplastic thyroid carcinoma, osteoarthritis, cardiac hypertrophy, myocardial infarction, chronic kidney diseases, and brain and cardiac infections.

## 5 Discussion

Small extracellular vesicles contain various classes of lipids, including phosphatidylserine, phosphatidylcholine (PC), phosphatidylinositol (PI), phosphatidylethanolamines (PE), ceramides, gangliosides, sterol lipids, sphingomyelin, and cholesterol (Llorente et al., 2013). Lipid profiles of sEVs are important as they contribute to fusion of sEVs with recipient cells (Donoso-Quezada et al., 2021). The composition of these lipid profiles is altered in many types of chronic diseases, including cancer, and enable them to be used as biomarkers (Blandin et al., 2023; Perpiñá-Clérigues et al., 2024). For instance, in ovarian cancer cells derived sEVs, there is an increase in zymosterol, gangliosides-3, lyso derivatives of PS, PC, phosphatidylglycerol (PG), and PI along with cholesterol esters (Cheng et al., 2020). Similar elevation of sEVs lipid composition is also observed in colorectal cancer (elevated levels of sphingolipids, glycolipids and sterols) and hepatocellular carcinoma (elevated levels of Lyso derivatives of PS, PI, PG, cardiolipins and saturated fatty acids (Elmallah et al., 2022; Sanchez et al., 2021).

Alternations in lipid composition also serve a role in an immune evasion. sEVs derived from cancer cells can modulate immune cells in the tumor microenvironment. The CD36 receptor mediates the uptake of fatty acids from sEVs by macrophages in the TME, influencing lipid metabolism within these cells and preventing them from activating CD8^+^ T cells. (Yang et al., 2022).

sEVs also modulate lipid metabolism in cancer by upregulating X-inactive specific transcript (XIST), which suppresses miR-655 and activates downstream signaling pathways that increase ATP citrate lyase (ACLY) expression. Additionally, sEVs enhance lipolysis in cancer-associated adipocytes, supplying energy and nutrients to support tumor growth. Cancer associated fibroblasts (CAF) secrete sEV containing acetates to promote formation of acetyl-coA, precursor of *de novo* fatty acid synthesis (Zhao et al., 2016). In addition, CAF derived sEVs aid in shunting carbon skeletons used in glycolytic pathway to reductive glutamine formation. This results in increased availability of citrate for use by ACLY, the rate limiting enzyme in fatty acid synthesis.

The Warburg effect contributes to decreased availability of acetyl-CoA as lactic acid is produced in hypoxia instead and sEVs aid in lipid deposition in cancer cells (Zhao et al., 2016). Cargos delivered by cancer cells can also promote beta oxidation instead of *de novo* fatty acid synthesis. This occurs in correlation with cancer-associated cachexia, which provides lots of free fatty acids that can be used by cancer cells to obtain energy via fatty acid oxidation pathways. Cancer cachexia is a syndrome characterized by a continuous loss of muscle mass, with or without loss of adipose tissue, resulting from a negative energy balance caused by the rapid proliferation of tumor cells. EVs from tumor cells can promote cachexia by fatty acid oxidation, which lead to negative impacts on cancer treatment (Hu X et al., 2021).

Moreover, changes in sEVs lipid composition may trigger lipid-associated cell death, such as ferroptosis (Liu et al., 2024; Reinicke et al., 2022). Ferroptosis is a type of programmed cell death mediated by iron accumulation and lipid peroxidation. Under oxidative stress, parent cells tend to secrete EVs containing antioxidants which have a protective role in recipient cells (Yarana et al., 2022). However, when parent cells have undergone ferroptosis, the EVs it releases promote ferroptosis associated pathways in recipient cells (Alarcón-Veleiro et al., 2023). Iron has also been identified as an important regulator of cell plasticity particularly for tumors and immune cells. Cancer stem cells have a high demand for iron uptake and storage. However, under oxidative stress, ferritin degradation occurs in lysosomes, leading to increased free iron levels. This has paved the way for use of ferroptosis inducers engineered as part of EVs to kill cancer cells alongside delivery of chemotherapeutic drugs (Liu et al., 2025).

Cancer-derivate EVs show alternations in their membrane lipid composition, indicating their potential as biomarker and therapeutic targets. While EV lipid composition is essential for vesicular stability and function, the direct effects of NCs on EVs lipid panel remain unexplored. Most of EVs’ studies about NCs describe its impact on vesicular cargo, such as proteins and miRNAs, rather than on lipids or lipid composition. However, numerous NCs demonstrate regulatory activity on enzymes essential for lipid synthesis and metabolism, such as sphingomyelinases, ceramide synthases, and ceramidases. These enzymes are directly involved in vesicular biogenesis, suggesting their potential to influence vesicular lipid composition. Therefore, future research should not be limited to the analysis of EV-associated nucleic acid and protein. Expanding the scope of EV studies to lipid panel after NCs treatment may reveal novel mechanisms on intercellular communication and cancer progression.

Future studies should integrate lipidomics analysis with protein and miRNA studies on NCs’ effects on EVs. Moreover, studies on how lipid alteration affects EV release and uptake in cancer cells could reveal new therapeutic directions (Azparren-Angulo et al., 2024). Together, these approaches may identify the lipid role in cancer progress and contribute to the development of lipid-targeted, EV-based interventions in oncology.

## 6 Conclusion

Small extracellular vesicles dominate oncogenesis by altering the tumor microenvironment and promoting metastasis. Oncogenic cells release sEVs with a unique composition specific to each cancer type and it play a key role in intercellular communication between cancer cells. They are useful biomarkers that help monitor cancer progression and prognosis non-invasively. Meanwhile, NCs like Manumycin A, Cannabidiol, Resveratrol, Honokiol and Dihydroartemisinin have mechanism of action that strongly correlates with cancer-derivate sEVs biogenesis and composition, making them a potential candidate in cancer treatment. Lipid as the fundamental structural unit of EV membranes, are essential for vesicle formation. Some NCs have been shown to interfere with lipogenesis, leading to imbalanced lipid homeostasis and indirectly affecting vesicular formation. Together, combination of NCs and sEV lipid profiling is a promising direction for cancer research.
